# Transcriptional Responses of Copper-Transport-Related Genes *ctr1*, *ctr2* and *atox1* and Their Roles in the Regulation of Cu Homeostasis in Yellow Catfish *Pelteobagrus fulvidraco*

**DOI:** 10.3390/ijms232012243

**Published:** 2022-10-13

**Authors:** Hong Yang, Chongchao Zhong, Xiaoying Tan, Guanghui Chen, Yang He, Shengzan Liu, Zhi Luo

**Affiliations:** 1 Hubei Hongshan Laboratory, Fishery College, Huazhong Agricultural University, Wuhan 430070, China; 2 Laboratory for Marine Fisheries Science and Food Production Processes, Qingdao National Laboratory for Marine Science and Technology, Qingdao 266237, China

**Keywords:** Cu, Cu transport, Cu homeostasis, transcriptional regulation, vertebrates

## Abstract

Here, we characterized the function of *ctr1*, *ctr2* and *atox1* promoters in yellow catfish *Pelteobagrus fulvidraco*, a common freshwater teleost in Asian countries. We obtained 1359 bp, 1842 bp and 1825 bp sequences of *ctr1*, *ctr2* and *atox1* promoters, and predicted key transcription factor binding sites on their promoters, including MRE, SREBP1, NRF2, KLF4 and STAT3. Cu differentially influenced the activities of *ctr1*, *ctr2* and *atox1* promoters from different regions. We found that the −326/−334 bp and −1232/−1240 bp locus in the *atox1* promoter were functional NRF2 binding sites, which negatively controlled the activity of the *atox1* promoter. The −91/−100 bp locus in the *ctr1* promoter and −232/−241 bp and −699/−708 bp locus in the *atox1* promoter were functional SREBP1 binding sites, which positively controlled the activities of *ctr1* and *atox1* promoters. Cu inhibited the NRF2 binding ability to the *atox1* promoter, but promoted the SREBP1 binding ability to the *ctr1* and *atox1* promoters. Dietary Cu excess significantly down-regulated hepatic mRNA and total protein expression of CTR1, CTR2 and ATOX1 of yellow catfish, compared to the adequate dietary Cu group. The subcellular localization showed that CTR1 was mainly localized on the cell membrane, CTR2 in the cell membrane and the lysosome, and ATOX1 in the cytoplasm. In conclusion, we demonstrated the regulatory mechanism of three Cu transporters at the transcription levels, and found the functional NRF2 and SREBP1 response elements in *ctr1*, *ctr2* and *atox1* promoters, which provided new insights into their roles in the regulation of Cu homeostasis in fish.

## 1. Introduction

Copper (Cu) is an essential mineral in vertebrates, including fish, and plays important roles in different metabolic pathways, including cellular respiration, transcriptional regulation, ion uptake and signal recognition [1,2]. However, when the body ingests excessive Cu, it will be potentially toxic [2]. Therefore, Cu homeostasis must be regulated tightly, which helps to maintain intracellular Cu levels within a reasonable range and prevent Cu toxicity.

The regulatory mechanism for Cu homeostasis is highly conserved from unicellular yeast to mammals, which was mainly involved in Cu uptake, distribution, storage and export [3,4]. Cu homeostasis is primarily controlled by several Cu-uptake-related proteins, such as copper-transport-related proteins (CTR1 and CTR2), two Cu-ATPase proteins (ATP7A and ATP7B) and three copper chaperone proteins (ATOX1, CCS and COX17) [5]. Among these members, CTR1, CTR2 and ATOX1 play important function in the regulation of Cu homeostasis. CTR1 is a highly active Cu transporter and mainly localized in the cytoplasmic membrane, which can transport Cu into cells in a low-Cu environment [3,6]. CTR2 is a low-affinity Cu transporter and more localized on the membrane of intracellular organelles, which can transport Cu when the environmental Cu concentration is high [7]. ATOX1 is one metallochaperone that delivers Cu to ATP7A and ATP7B transporters [8]. Currently, the functions of these Cu transporter proteins have been studied [8,9,10], but their transcriptionally regulatory mechanisms in vertebrates, including fish, are unclear.

In eukaryotes, studies suggested that the most effective regulation for gene expression occurs at the transcriptional level [11]. Therefore, it is very crucial to study how the genes initiate their transcription. Nuclear factor erythroid 2-related factor 2 (NRF2) is an important regulator of the body’s antioxidative stress, which can bind to antioxidant response elements (AREs, 5’-TGACNNGC-3’) to activate the transcription of downstream genes [12]. The sterol regulatory element-binding protein 1 (SREBP1) coordinates the gene transcription of enzymes involved in the lipogenic pathway [13,14]. Studies suggested that Cu enhanced NRF2 signaling [15,16] and increased the *srebp1* mRNA expression [14,17]. At present, although the function and its potential targets of NRF2 and SREBP1 have been studied [18,19], it remains unknown whether these transcriptional factors mediated the regulation of these Cu transporters. As a matter of fact, it is of great significance to study whether NRF2 and SREBP1 can directly target Cu-transport-related genes to regulate Cu homeostasis.

In the present study, we used yellow catfish *Pelteobagrus fulvidraco*, a common freshwater teleost in China and other Asian countries, as the experimental fish because its genome information is available openly, and because yellow catfish had physiological processes similar to other vertebrates [20]. Recently, we have isolated and characterized the *ctr1*, *ctr2* and *atox1* full-length cDNA sequences in yellow catfish [5]. To further investigate their functions and the regulatory mechanism of Cu transporters, this study characterized *ctr1*, *ctr2* and *atox1* promoters in yellow catfish, and demonstrated the transcriptionally regulatory mechanism of these three Cu-transport-related proteins in response to Cu, and determined their intracellular localization.

## 2. Results

### 2.1. Sequence Analysis of the ctr1, ctr2 and atox1 Promoters

We cloned the 1359 bp *ctr1* promoter (Figure S1), the 1842 bp *ctr2* promoter (Figure S2) and the 1825 bp *atox1* promoter (Figure S3) in yellow catfish. We also identified the transcription start sites (TSS) of *ctr1, ctr2* and *atox1* promoters. The first nucleotide of each TSS was designated as +1. Some core promoter elements were predicted, including TATA-box (TBP) at −47/−68 bp of the *ctr1* promoter, −23/−37 bp of the *ctr2* promoter and −131/−145 bp of the *atox1* promoter; CCAAT-box (NF-Y) at −126/−136 bp of the *ctr1* promoter; and GC-box (SP1) at −105/−115 bp of the *ctr1* promoter, −430/−439 bp and −1243/−1250 bp of the *ctr2* promoter and −248/−257 bp and −1785/−1795 bp of the *atox1* promoter. Several putative TFBSs were predicted on the *ctr1, ctr2* and *atox1* promoters. On the *ctr1* promoter, we predicted a series of TFBSs, such as MRE (−13/−25 bp, −621/−634 bp and −1233/−1247 bp), SREBP1 (−91/−100 bp, −114/−123 bp and −664/−673 bp), AP1 (−295/−302 bp), STAT3 (−693/−706 bp), MAC1 (−730/−737 bp, −765/−772 bp and −1220/−1227 bp), NRF2 (−820/−830 bp, −1050/−1059 bp), CREB1 (−1015/−1026 bp) and KLF4 (−1279/−1289 bp). In the *ctr2* promoter, a cluster of TFBSs was predicted, including peroxisome-proliferator-activated receptor gamma (PPARγ) (−112/−126 bp and −1076/−1080 bp), CREB1 (−166/−173 bp and −1364/−1376 bp), AP1 (−352/−362 bp), STAT3 (−537/−547 bp), SREBP1 (−630/−639 bp and −1058/−1067 bp), STAT4 (−904/−917 bp), MAC1 (−1390/−1396 bp and −1540/−1547 bp) and KLF4 (−1570/−1580 bp). In the *atox1* promoter, several important TFBSs, such as NRF2 (−37/−47 bp, −326/−334 bp and −1232/−1240 bp), STAT4 (−66/−80 bp), STAT3 (−177/−187 bp), SREBP1 (−232/−241 bp and −699/−708 bp), AP1 (−363/−373 bp, −936/−944 bp and −1655/−1662 bp), KLF4 (−727/−736 bp and −748/−757 bp), CREB1 (−1182/−1194 bp and −1427/−1439 bp) and MAC1 (−1063/−1070 bp), were also found.

### 2.2. Analysis of the 5′-Sequence Deletion of the ctr1, ctr2 and atox1 Promoters

**Figure 1:**
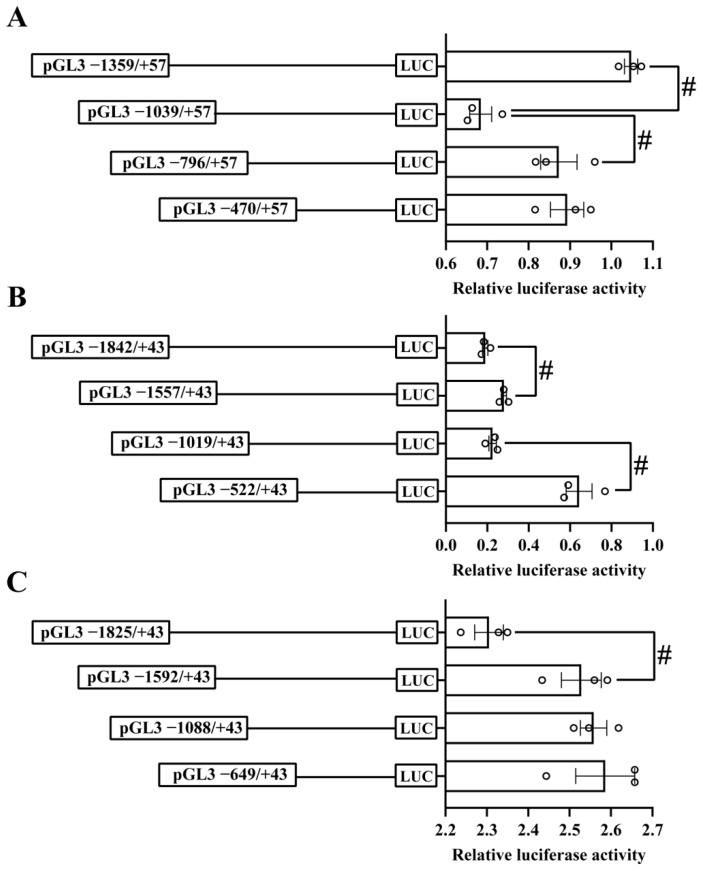
5′ unidirectional deletion assays of *ctr1* (**A**), *ctr2* (**B**) and *atox1* (**C**) promoters of yellow catfish. Values mean the ratio of activities of Firefly to Renilla luciferase. Results are presented as means ± SEM (n = 3). Hash symbol (#) means significant differences between two groupss (*p* < 0.05).

We constructed the 5′-sequence deletion plasmids and transfected them into the HEK293T cells to identify the core regions of three promoters (Figure 1). For the *ctr1* promoter, the sequence deletion from −796 bp to −1039 bp significantly reduced the relative luciferase activity, but the sequence deletion from −1039 bp to −1359 bp significantly increased the relative luciferase activity (Figure 1A). For the *ctr2* promoter, the sequence deletion from −522 bp to −1019 bp and from −1557 bp to −1842 bp significantly reduced the luciferase activities (Figure 1B). For the *atox1* promoter, the luciferase activity of the sequence deletion from −1592 bp to −1825 bp was significantly reduced (Figure 1C).

**Figure 2:**
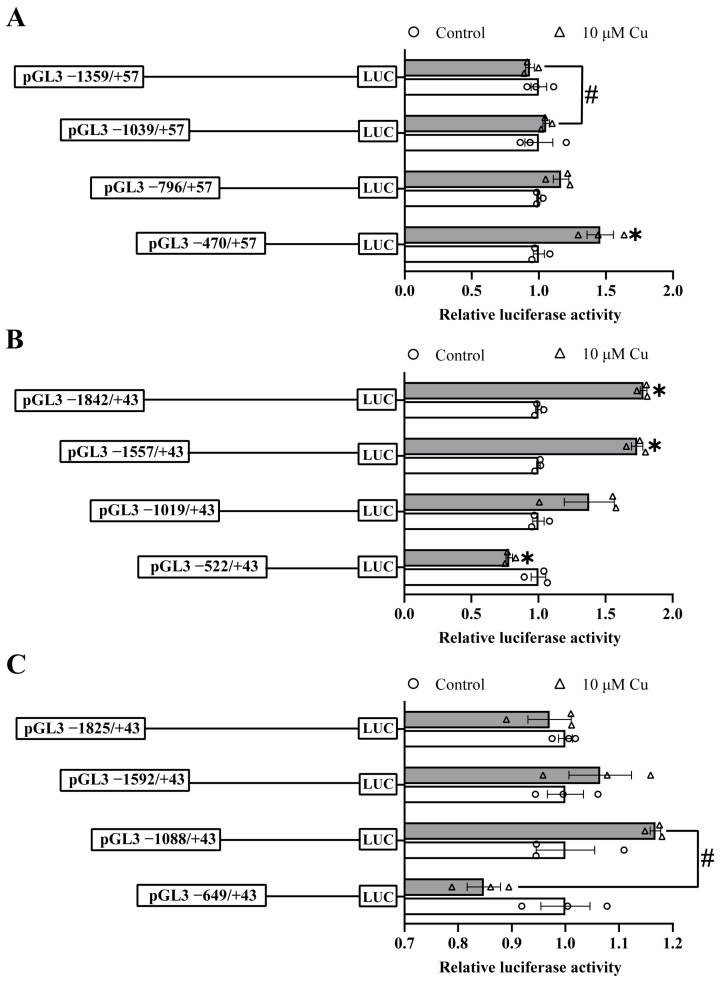
5′ unidirectional deletion assays for *ctr1* (**A**), *ctr2* (**B**) and *atox1* (**C**) promoters after 10 μM Cu incubation. Values show the ratio of activities of Firefly to Renilla luciferase, normalized to the control, and are presented as means ± SEM (n = 3). Asterisk (*) indicates significant differences in relative luciferase activities between the Cu-treated group and the control in the same fragment (*p* < 0.05); hash symbol (#) indicates significant differences in relative luciferase activities between the two fragmentss under the same treatment (*p* < 0.05).

We also used 5’-sequence deletion analysis to explore the response of these three promoters to Cu (Figure 2). For the *ctr1* promoter, compared with the control, Cu treatment increased the relative luciferase activity of the −470/+57 bp sequence, indicating the regulatory effect of Cu on the *ctr1* promoter. For different sequence deletion plasmids treated with Cu, the luciferase activity of the −1039/+57 bp region was higher than that of −1359/+57 bp, suggesting that the region from −1039 bp to −1359 bp has a negatively regulatory element (Figure 2A). For the *ctr2* promoter, compared with the control, Cu treatment reduced the relative luciferase activity of the −522/+43 bp sequence but increased the luciferase activities of −1557/+43 bp and −1842/+43 bp sequences, indicating both positive and negative regulatory elements in response to Cu on the *ctr2* promoter (Figure 2B). For the *atox1* promoter, the activity of the sequence deletion from −649 bp to −1088 bp was significantly up-regulated after Cu treatment, indicating the positively regulatory element in this region (Figure 2C).

### 2.3. Site-Mutation Analysis of the NRF2 and SREBP1 Binding Sites on ctr1, ctr2 and atox1 Promoters 

**Figure 3:**
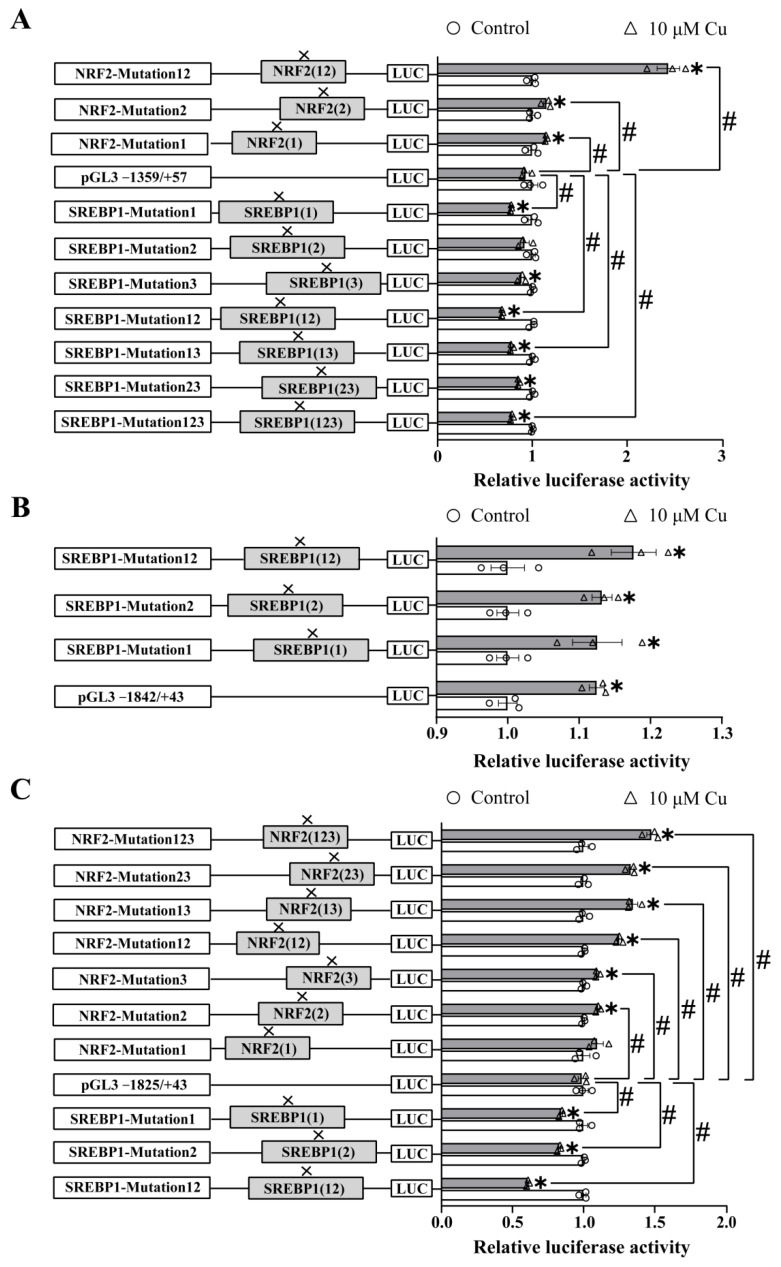
Assays of predicted NRF2 and SREBP1 binding sites of yellow catfish *ctr1*, *ctr2* and *atox1* promoters after site-directed mutagenesis. (**A**) Site mutagenesis of NRF2 and SREBP1 on pGl3-ctr1-1359 vector. (**B**) Site mutagenesis of SREBP1 on pGl3-ctr2-1842 vector. (**C**) Site mutagenesis of NRF2 and SREBP1 on pGl3-atox1-1825 vector. Values mean the ratio of activities of Firefly to Renilla luciferase, normalized to the control. Results are presented as means ± SEM (n = 3). Asterisk (*) indicates significant differences between the Cu-treated group and the control in the same fragment (*p* < 0.05); hash symbol (#) indicates significant differences in relative luciferase activities between the two fragments under the same treatment (*p* < 0.05).

We verified the potential TFBSs of *ctr1*, *ctr2* and *atox1* promoters through the site-mutation analysis (Figure 3). For the *ctr1* promoter, compared with the control, the mutation of −820/−830 bp NRF2 site (Mut-ctr1-NRF2(1)), −1050/−1059 bp NRF2 site (Mut-ctr1-NRF2(2)) and the combined mutation of these two sites (Mut-ctr1-NRF2(12)) increased the luciferase activities, but the −91/−100 bp SREBP1 site (Mut-ctr1-SREBP1(1)), −664/−673 bp SREBP1 site (Mut-ctr1-SREBP1(3)) mutant fragments and fragments (Mut-ctr1-SREBP1(12), Mut-ctr1-SREBP1(13), Mut-ctr1-SREBP1(23) and Mut-ctr1-SREBP1(123)) containing these two mutant sites reduced luciferase activities, indicating that the NRF2 sites played negative regulatory roles but the SREBP1(1) site and the SREBP1(3) site played positive regulatory roles in the *ctr1* promoter. When HEK293T cells were incubated with Cu, compared to the pGl3 −1359/+57 vector, the mutation of the −820/−830 bp NRF2 site (Mut-ctr1-NRF2(1)), the −1050/−1059 bp NRF2 site (Mut-ctr1-NRF2(2)) and the combined mutation of these two sites alleviated the Cu-induced decrease in luciferase activities. However, the mutation of −91/−100 bp SREBP1 site (Mut-ctr1-SREBP1(1)) and these fragments (Mut-ctr1-SREBP1(12), Mut-ctr1-SREBP1(13) and Mut-ctr1-SREBP1(123)) containing this mutant site aggravated the Cu-induced decrease in luciferase activities. These indicated that the two NRF2 sites had negative regulatory roles in response to Cu, and the SREBP1(1) site had a positive regulatory role in response to Cu (Figure 3A).

For the *ctr2* promoter, no potential NRF2 binding sites were predicted in the present study. Compared with the control, with the Cu treatment, the mutation of the −630/−639 bp SREBP1 site (Mut-ctr2-SREBP1(1)) and the −1058/−1067 bp SREBP1 site (Mut-ctr2-SREBP1(2)) increased the luciferase activities. Therefore, the plasmid (Mut-ctr2-SREBP1(12)) containing these two mutation sites also up-regulated luciferase activity under the Cu treatment, indicating that the SREBP1 binding sites were important for Cu-induced transcriptional regulation of the *ctr2* promoter (Figure 3B).

For the *atox1* promoter, after Cu treatment, mutations at the −326/−334 bp NRF2 site (Mut-atox1-NRF2(2)) and −1230/−1240 bp NRF2 site (Mut-atox1-Nrf2(3)) and plasmids (Mut-atox1-NRF2(12), Mut-atox1-NRF2(13), Mut-atox1-NRF2(23) and Mut-atox1-NRF2(123)) containing these two mutation sites up-regulated luciferase activities. Conversely, mutations at the −232/−241 bp SREBP1 site (Mut-atox1-SREBP1(1)) and −699/−708 bp SREBP1 site (Mut-atox1-SREBP1(2)) and plasmid (Mut-atox1-SREBP1(12)) mutated at these two sites down-regulated luciferase activities. Similarly, compared to the pGl3 −1359/+57 vector, the mutation of the −326/−334 bp NRF2 site (Mut-atox1-Nrf2(2)) and −1232/−1240 bp NRF2 site (Mut-atox1-NRF2(3)) and plasmids (Mut-atox1-NRF2(12), Mut-atox1-NRF2(13), Mut-atox1-NRF2(23) and Mut-atox1-NRF2(123)) containing these two mutation sites increased luciferase activities. Additionally, the mutation of the −232/−241 bp SREBP1 site (Mut-atox1-SREBP1(1)) and −699/−708 bp SREBP1 site (Mut-atox1-SREBP1(2)) and the combined mutation of these two sites (Mut-atox1-SREBP1(12)) reduced luciferase activities, suggesting that the NRF2(2) site and NRF2(3) site played negative regulatory roles but the two SREBP1 sites played positive regulatory roles in Cu-induced transcriptional regulation of the *atox1* promoter (Figure 3C). 

### 2.4. Analysis of the Functional Binding Sites Based on EMSA

**Figure 4:**
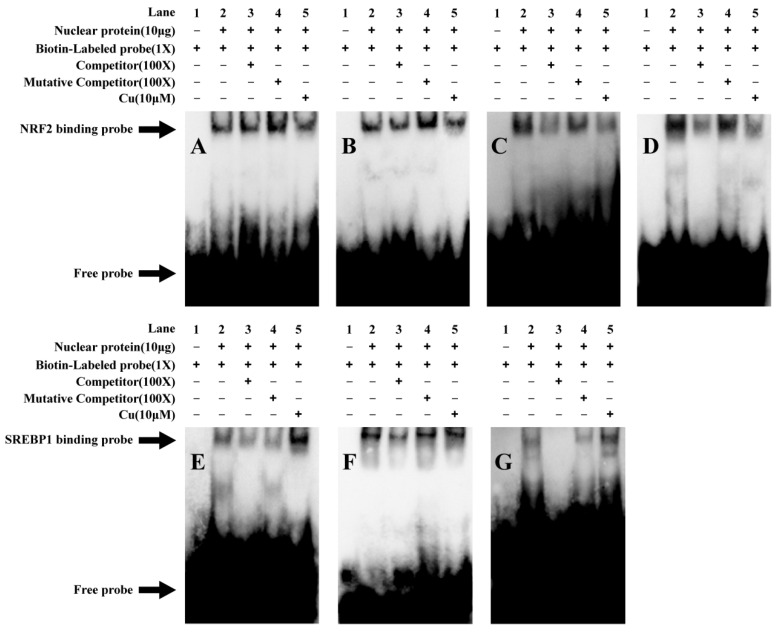
EMSA of predicted NRF2 and SREBP1 binding sequences on yellow catfish the *ctr1*, *ctr2* and *atox1* promoters. (**A**) NRF2 binding sequence sited between −820 bp and −830 bp of the *ctr1* promoter; (**B**) NRF2 binding sequence sited between −1050 bp and −1059 bp of the *ctr1* promoter; (**C**) NRF2 binding sequence sited between −326 bp and −334 bp of the *atox1* promoter; (**D**) NRF2 binding sequence sited between −1232 bp and −1240 bp of the *atox1* promoter; (**E**) SREBP1 binding sequence sited between −91 bp and −100 bp of the *ctr1* promoter; (**F**) SREBP1 binding sequence sited between −232 bp and −241 bp of the *atox1* promoter; (**G**) SREBP1 binding sequence sited between −699 bp and −708 bp of the *atox1* promoter; NP: nuclear protein. The numbers 1–5 represent the five different lanes.

Based on the above site mutation analysis, the −820/−830 bp and −1050/−1059 bp sites for NRF2 binding and the −91/−100 bp site for SREBP1 binding in the *ctr1* promoter, as well as the −326/−334 bp and −1232/−1240 bp sites for NRF2 binding and the −232/−241 bp and −699/−708 bp sites for SREBP1 binding in the *atox1* promoter, were considered functional. Thus, EMSA was used to further verify these potential binding sites (Figure 4). For the *ctr1* promoter, the 100-fold unlabeled NRF2 binding sequences (−820/−830 bp and −1050/−1059 bp) and the sequences (Mut-ctr1-NRF2(1) and Mut-ctr1-NRF2(2)) mutated from the 100-fold unlabeled NRF2 binding sites did not compete for the binding of the added protein (Lane 3–4), suggesting that NRF2 could not combine with these two regions. Compared with the control, Cu treatment did not significantly influence the band brightness, indicating that the NRF2 site of the *ctr1* promoter did not interact with Cu (Figure 4A,B). 

For the *atox1* promoter, the 100-fold unlabeled NRF2 binding site (−326/−334 bp and −1230/−1240 bp) competed for the binding (Lane 3), while the 100-fold unlabeled Mut-NRF2 sequences ((Mut-atox1-NRF2(2) and Mut-atox1-NRF2(3)) did not compete for the nuclear protein with the labeled probe (Lane 4). After Cu incubation, the brightness of the bands was weaker than that of the bands in the control, indicating that the −326/−334 bp and −1232/−1240 bp NRF2 binding sites on the *atox1* promoter could bind with the nuclear protein, and Cu incubation weakened the binding (Figure 4C,D).

Similarly, we used the SREBP1 binding sequences as the probes. For the *ctr1* promoter, the 100-fold unlabeled SREBP1 binding sequence (−91/−100 bp) competed for the nuclear protein with the labeled probe (Lane 3–4). Compared with the control, the brightness of the last band was significantly higher than the band brightness in the control, indicating that the −91/−100 bp SREBP1 sequence was a functional binding site and that Cu was involved in the transcriptional regulation of the *ctr1* promoter (Figure 4E). For the *atox1* promoter, the 100-fold unlabeled −232/−241 bp and −699/−708 bp SREBP1 binding sequences competed for the nuclear protein with the labeled probe (Lane 3), while the 100-fold unlabeled SREBP1 binding sites’ mutated sequences (Mut-atox1-SREBP1(1) and Mut-atox1-SREBP1(2)) did not compete for the binding, suggesting that SREBP1 could bind with these regions of the *atox1* promoter. Compared with the control, Cu increased the band brightness, suggesting that Cu mediated the transcriptional regulation of *atox1* promoter between the −232/−241 bp and −699/−708 bp SREBP1 binding sites (Figure 4F,G).

### 2.5. The Effects of Dietary Cu Levels on ctr1, ctr2 and atox1 mRNA and Protein Expressions in Yellow Catfish Liver Tissues

**Figure 5:**
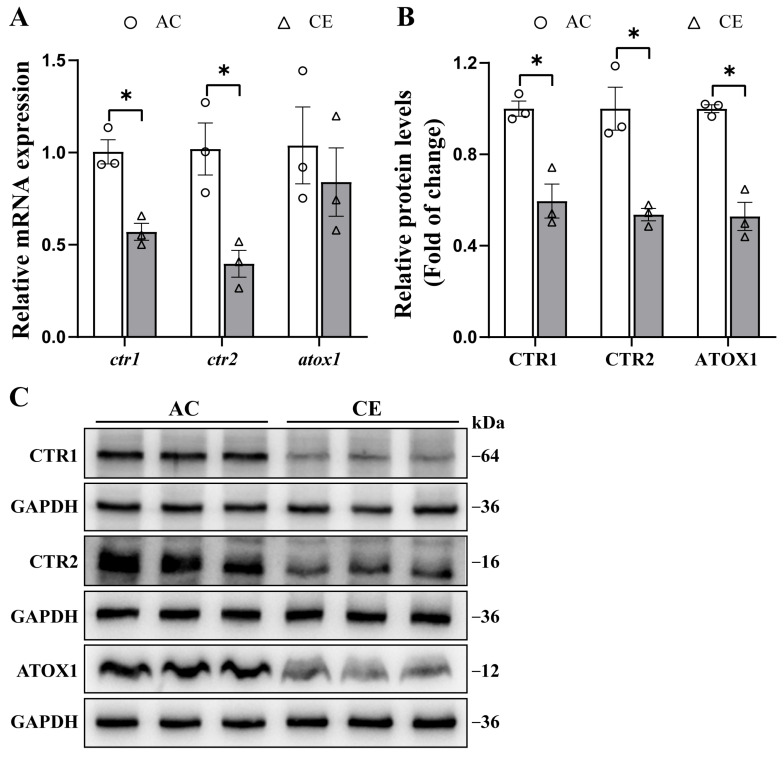
Effects of dietary Cu levels on *ctr1*, *ctr2* and *atox1* expression in the liver tissues of yellow catfish. (**A**) *ctr1*, *ctr2* and *atox1* gene expressions normalized to *tbp* and *rpl7*; (**B**) the relative densities of total CTR1, CTR2 and ATOX1 protein were measured by Image-Pro Plus; (**C**) CTR1, CTR2 and ATOX1 total protein expression. Values are means ± SEM (*n* = 3), and experiments were repeated three times. *p* value was calculated by one-way ANOVA and further post hoc Duncan’s multiple range testing. Asterisk (*) indicates significant differences between the two treatments (*p* < 0.05).

In order to study the effects of Cu on *ctr1*, *ctr2* and *atox1* mRNA and protein expressions, we designed in vivo experiments (Figure 5). The original western blot figures are given in File S1. Compared with the AC (adequate Cu) group, Cu excess down-regulated the *ctr1* and *ctr2* mRNA levels, but did not significantly influence the *atox1* mRNA level (Figure 5A). Cu expression also significantly down-regulated the CTR1, CTR2 and ATOX1 protein expressions (Figure 5B,C).

### 2.6. Subcellular Localization of CTR1, CTR2 and ATOX1 in HEK293T Cells

**Figure 6:**
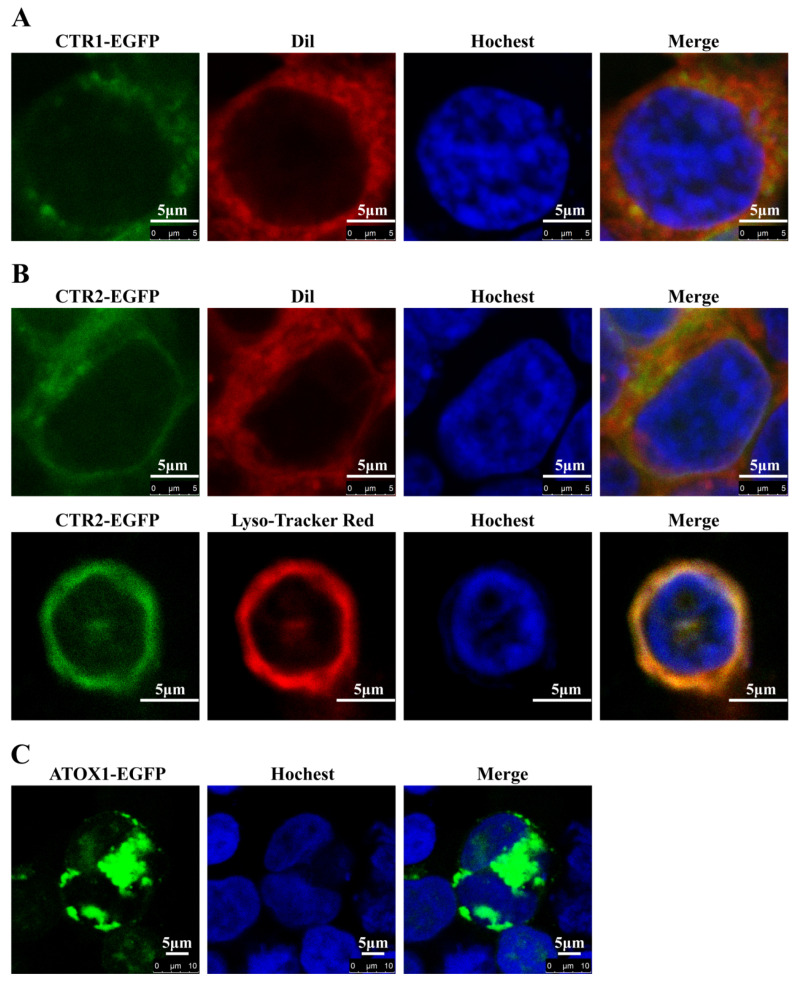
Subcellular localization of yellow catfish CTR1, CTR2 and ATOX1. (**A**) Subcellular localization of CTR1; (**B**) Subcellular localization of CTR2; (**C**) Subcellular localization of ATOX1. CTR1-EGFP, CTR2-EGFP and ATOX1-EGFP were transfected into the HEK293T cells. Cell membrane, nucleus and lysosome were stained with Dil, Hochest and Lyso-Tracker Red, respectively. A laser scanning confocal microscope was used to visualize the localization.

To determine the intracellular localization of CTR1, CTR2 and ATOX1 in yellow catfish, we transfected fusion CTR1-EGFP, CTR2-EGFP and ATOX1-EGFP plasmids into HEK293T cells (Figure 6). The original subcellular localization images are given in File S1. The results showed that CTR1 was mainly located in the cell membrane (Figure 6A), CTR2 in the cell membrane and the lysosome (Figure 6B). ATOX1 was widely expressed in the cytoplasm rather than in the nucleus (Figure 6C).

## 3. Discussion

CTR1, CTR2 and ATOX1 play important roles in controlling intracellular Cu homeostasis and are regulated by Cu [5,9], but the mechanism remains unclear. Here, we characterized the function and transcriptional regulation of *ctr1*, *ctr2* and *atox1* genes in response to Cu in yellow catfish, which provided new insights into their roles in the control of Cu homeostasis.

The identification of core promoters is the first step in investigating the mechanism of transcription initiation [21]. Here, we predicted core elements on the *ctr1*, *ctr2* and *atox1* promoters of yellow catfish, including a GC box (SP1), TATA-box and CCAAT-box (NF-Y) in the *ctr1* promoter, similar to other reports [22,23], and a TATA-box on the *ctr2* promoter, similar to this report by Beneš et al. [24]. We also predicted two SP1s in the *ctr2* and *atox1* promoters, respectively, but it was unclear whether SP1 was involved in the control of the *ctr2* and *atox1* promoters [25,26]. However, we found neither CCAAT-box nor TATA-box in the core region of the *atox1* promoter. Similarly, Roy and Singer [27] reported that only about 5–7% of eukaryotic promoters had the TATA-box. We also predicted a cluster of the TFBSs, such as NRF2, SREBP1, MAC1, STAT3, STAT4, KLF4, PPARγ, AP1 and CREB1, in the three gene promoters. Similar TFBSs were also found in other promoters [15,28]. However, it is unclear whether these functional TFBSs exist in the *ctr1*, *ctr2* and *atox1* promoters of other species. 

Since NRF2 binding sites existed on *ctr1* and *atox1* promoters, and SREBP1 binding sites existed on all three promoters, we explored whether they were functional sites. In the present study, site mutation analysis showed that NRF2 significantly reduced the luciferase activities of *ctr1* and *atox1* promoters. NRF2 is a transcription factor that potently transduces chemical signals to regulate a range of cytoprotective genes [18]. Our result suggested that NRF2 repressed the expression of downstream *ctr1* and *atox1* genes. Furthermore, we found that Cu incubation exacerbated the down-regulation of relative luciferase activities by NRF2 on the *ctr1* and *atox1* promoters, suggesting that NRF2 inhibited the expressions of the downstream *ctr1* and *atox1* genes more obviously under Cu incubation. Similarly, Zeng et al. [16] found that in the anterior and mid-intestines of large yellow croaker, Cu stress up-regulated the mRNA expressions of *nrf2* and down-regulated the mRNA expressions of *ctr1*, indicating that there might be an antagonistic effect between NRF2 and CTR1 under Cu stress. SREBP1 is an important transcriptional regulator of lipogenesis [29]. Our study indicated that SREBP1 at the −91/−100 bp binding site of the *ctr1* promoter and SREBP1 at the −232/−241 bp and −699/−708 bp binding sites of the *atox1* promoter could significantly up-regulate the relative luciferase activities; Cu incubation further aggravated the up-regulation of SREBP1, suggesting that SREBP1 could promote the expressions of the downstream *ctr1* and *atox1* genes. Similarly, Dong et al. [30] found that the protein expressions of SREBP1 were enhanced in obese spontaneously hypertensive (SHROB) rats, and the induction of *atox1* mRNA was also higher in the liver of male rats, indicating the potentially synergistic effect between SREBP1 and ATOX1. We also predicted two SREBP1 binding sites (−114/−123 bp and −664/−673 bp) on the *ctr1* promoter and two SREBP1 binding sites (−630/−639 bp and −1058/−1067 bp) on the *ctr2* promoter, but the relative luciferase activities did not change significantly after these SREBP1 binding sites were mutated, suggesting that these four sites were not functional. Thus, our results suggested that NRF2 and SREBP1 had distinct regulatory roles by targeting the *ctr1*, *ctr2* and *atox1* promoters in yellow catfish. Zhong et al. [31] demonstrated that in the liver of yellow catfish, the mRNA levels of *srebp1* did not change significantly when compared with the adequate Cu group, while the NRF2 protein expression increased significantly in the Cu excess group. Similarly, Yu et al. [32] also found that juvenile blunt snout bream *Megalobrama amblycephala* had lower hepatic *srebp1* mRNA expressions but higher mRNA levels of *nrf2* after feeding 0.04% fenugreek seed extracts (FSE) diets. 

In our study, although site mutation analysis indicated that NRF2 caused changes in the luciferase activities of the *ctr1* promoter under Cu treatment, EMSA analysis showed that the 100-fold unlabeled NRF2 binding sequences (−820/−830 bp and −1050/−1059 bp) did not compete for the binding of the added protein. Thus, we speculated that these two NRF2 binding sites were also not functional. Battino et al. [33] showed that after NRF2 is translocated to the nucleus, it forms complexes with coactivators and binds to promoter regions (AREs) to activate the expressions of target genes. Therefore, we thought that NRF2 regulated the *ctr1* promoter by combining with other coactivators to form a complex. In addition, our study indicated that the −326/−334 bp and −1230/−1240 bp NRF2 binding sequences of *atox1* promoter competed for the binding, suggesting that the positions from −326 bp to −334 bp and −1230 bp to −1240 bp were functional binding sites and Cu inhibited the binding of nuclear protein to the sites. At present, no research proved the direct regulatory effects of NRF2 on the *ctr1* and *atox1* promoters. We also found that the −91/−100 bp SREBP1 binding sequence of *ctr1* promoter and −232/−241 bp and −699/−708 bp SREBP1 binding sequences of *atox1* promoter competed for binding, indicating that these three sites were functional sites, and Cu facilitated the binding of nuclear protein to these sites. Pan et al. [14] showed that Cu significantly increased the gene expression of *srebp1* in the hepatocytes of zebrafish. Huang et al. [34] pointed out that hepatic *srebp1* mRNA levels were significantly increased in *Synechogobius hasta* when exposed to waterborne Cu for 30 days, whereas *srebp1* mRNA levels were significantly decreased after 60 days of exposure. Our research also suggested that NRF2 indirectly mediated transcriptional activity of *ctr1* but directly mediated transcriptional activity of *atox1*, and that SREBP1 directly mediated transcriptional activities of *ctr1* and *atox1*. NRF2 are the member of the basic leucine zipper family [15]. At present, whether NRF2 directly associates with Cu-transport-related proteins to regulate Cu homeostasis remains unknown in fish. SREBP1 is a transcription factor that regulates the expressions of enzymes required for endogenous cholesterol [14]. Furthermore, Cu-induced changes in SREBP1 levels have been reported in several studies. For instance, Tang et al. [35] suggested that Cu deficiency stimulated hepatic lipogenic gene expressions by increasing the hepatic translocation of mature SREBP1. Other studies indicated that the Cu-induced effects on *srebp1* mRNA levels were fish species- and tissue-dependent [14,17,36]. However, to our knowledge, the present study is the first report on the presence of SREBP1 sites on *ctr1*, *ctr2* and *atox1* promoters. Our results indicated that Cu had different regulatory effects on *ctr1*, *ctr2* and *atox1* promoters of different lengths through NRF2 and SREBP1 transcription factors, thereby maintaining Cu homeostasis.

Cu-transport-related proteins function in modulating Cu homoeostasis, but the mechanisms by which these proteins regulate Cu levels remain to be further investigated [9]. Therefore, we cultured yellow catfish with two dietary Cu levels to explore the regulation of Cu levels and Cu-transport-related proteins expressions. Our study found that mRNA and protein levels of *ctr1* and *ctr2* in the liver of yellow catfish were significantly down-regulated in the CE (Cu excess) group, indicating that the expression of *ctr1* and *ctr2* was inhibited under high dietary Cu levels. Our previous study [5] showed that the *ctr1* mRNA levels were significantly down-regulated in the anterior and mid-intestine of yellow catfish when Cu was excessive, while the *ctr2* mRNA levels were significantly up-regulated in the anterior intestine but down-regulated in the mid-intestine. Similarly, another study reported that Cu reduced mRNA levels of *ctr1* in the gill of guppy *Poecilia vivipara* [37]. Therefore, these results suggested that the expression levels of *ctr1* and *ctr2* would be reduced in the presence of Cu excess to maintain Cu homeostasis and to guard against Cu toxicity. Our study also found that dietary Cu levels did not influence the mRNA levels of *atox1* in the liver of yellow catfish but Cu excess reduced its protein levels, indicating that the ATOX1 was regulated by Cu at the protein levels. In contrast, Cheng et al. [5] found that the *atox1* mRNA levels were significantly down-regulated in the anterior intestine and mid-intestine of yellow catfish under Cu excess. Zhao et al. [38] found that during Cu^2+^ exposure, red swamp crayfish inhibited intracellular Cu transport by suppressing the expression levels of *ctr1* and *atox1* in the hepatopancreas. The results showed that ATOX1 acts as a Cu chaperone, and its expression was tightly regulated by intracellular Cu concentration, thereby preventing excessive Cu-induced high cytotoxicity and maintaining intracellular Cu homeostasis. Cu homeostasis is ensured by the activities of metal transporters and intracellular chaperones [9,39]. Loss of ATOX1 has been reported to result in impaired intracellular Cu efflux, significantly increased intracellular Cu content, and abnormal intracellular Cu distribution [40,41]. However, the mechanism by which Cu transporters in fish were affected by Cu concentration was still unclear and needed to be further explored. 

In this study, CTR1 was mainly located in the cell membrane. Ohrvik and Thiele [4] suggested that the localization of CTR1 on the cell membrane can form a highly selective ion channel structure to transport extracellular cuprous ions (Cu^+^) into the cells. We found that CTR2 was located in the cell membrane and the lysosome. Studies had demonstrated that CTR2 was more localized on the membrane of intracellular organelles, such as vacuole, membrane vesicle, endosome and lysosome, in addition to its localization on the cell membrane [7]. CTR2 located on lysosomes can transport Cu ions from organelles to the cytosols, which may have a role in recycling Cu from intracellular reservoirs. CTR2 localized on the cell membrane plays the same role as CTR1, participating in Cu uptake. Bertinato et al. [42] showed that after over-expression of CTR2 in Cu-deficient COS-7 cells, culture under Cu-rich conditions resulted in excessive accumulation of cellular Cu. Moreover, our study found that ATOX1 was localized in the cytoplasm. Chen et al. [8] also found that ATOX1 was mainly localized in the cytoplasm when it was not functioning. ATOX1 plays a crucial role in cellular Cu homeostasis. ATOX1 captures Cu in the cytosols for subsequent transfer to Cu pumps in the anti-Golgi network, thereby facilitating the supply of Cu to various Cu-dependent oxidoreductases that mature in secretory vesicles [43]. In addition, ATOX1 functioned as a Cu-chaperone for CRIP2, a nuclear copper-dependent autophagy activator [43]. Therefore, these results could reflect the roles of CTR1, CTR2 and ATOX1 in maintaining Cu homeostasis, respectively. 

## 4. Materials and Methods

### 4.1. Ethical Statement

The experimental protocols performed in yellow catfish followed the guideline of the Ethics Committee of Huazhong Agricultural University (HZAU) for the use of experimental animals and were approved by the Ethics Committee of HZAU. 

### 4.2. Experimental Animals, Cells and Reagents

In order to clone the *ctr1*, *ctr2* and *atox1* promoters and characterize their function, yellow catfish were purchased from a local farm (Wuhan, China). HEK293T cells were obtained from the Cell Resource Center in the Fishery College of HZAU. The Lipofectamine 293 and LightShift Chemiluminescent EMSA Kit were purchased from Beyotime Biotechnology (Shanghai, China); Passive Lysis Buffer and Dual-Luciferase from Promega (Minneapolis, MN, USA); and 0.25% trypsin-EDTA, Dulbecco’s Modified Eagle’s Medium (DMEM), Serum-Free Media, Serum-Free Cell Freezing Medium and fetal bovine serum (FBS) from Gibco (Waltham, MA, USA). The Pierce BCA protein assay kit, Trizol reagent and the Reverse Transcription Kit were from Thermo Fisher Scientific (Waltham, MA, USA).

### 4.3. Exp. 1: Identification and Functional Analysis of Ctr1, Ctr2 and Atox1 Promoters

#### 4.3.1. Promoter Identification and Plasmid Construction 

We identified the transcription start sites (TSS) of yellow catfish *ctr1*, *ctr2* and *atox1* genes according to our previous studies [28,44]. Then, we cloned their promoter sequences based on yellow catfish genome information [20] and on those in Xu et al. [44]. Using the Sac I and Hind III restriction sites, we subcloned various plasmids containing the sequences of *ctr1*, *ctr2* and *atox1* promoters into the pGl3-Basic vectors (Promega, Fitchburg, WI, USA). The ClonExpress II One Step Cloning Kit (Vazyme, Piscataway, NJ, USA) was utilized to connect the purified PCR product with the pGl3-Basic vectors to generate the luciferase reporter constructs. Based on their distances from the TSS, we defined these plasmids as pGl3 −1359/+57 of *ctr1,* pGl3 −1842/+43 of *ctr2* and pGl3 −1825/+43 of *atox1*. Then, the template pGl3 −1359/+57 of *ctr1* vector was used to generate the plasmids pGl3 −470/+57, pGl3 −796/+57 and pGl3 −1039/+57 of *ctr1*. Similarly, the plasmids pGl3 −522/+43, pGl3 −1019/+43 and pGl3 −1557/+43 of *ctr2*, and pGl3 −649/+43, pGl-1088/+43 and pGl3 −1592/+43 of *atox1* were obtained by the pGl3 −1842/+43 of *ctr2* vector and pGl3 −1825/+43 of *atox1* vector, respectively. Tables S1 and S2 list these primers for the promoter cloning and the plasmid construction.

#### 4.3.2. Sequence Analysis 

JASPAR database (http://jaspar.genereg.net/, accessed on 28 May 2021), TFSEARCH (http://www.cbrc.jp/research/db/TFSEARCH.html, accessed on 28 May 2021) and MatInspector (http://www.genomatix.de/, accessed on 28 May 2021) were used to predict the transcriptional factor binding sites (TFBSs) of *ctr1, ctr2* and *atox1* promoters. Then, the Clustal-W multiple alignment algorithm was utilized to perform the sequence alignments.

#### 4.3.3. Analysis of the 5′-Sequence Deletion of ctr1, ctr2 and atox1 Promoters 

According to the method of Xu et al. [44], we transfected the plasmids into HEK293T cells and measured their relative luciferase activities. Briefly, the HEK293T cells were cultured in the DMEM medium containing 10% (*v*/*v*) heat-inactivated FBS (Gibco, CA, USA) in an incubator at 37 °C and 5% CO_2_. Lipofectamine 293 (Beyotime Biotechnology, Shanghai, China) was used as the transfection reagent. Before transfection, the HEK293T cells were cultured in a 24-well plate at 1.2 × 10^5^ cells/well. They were cultured for 24 h to 70-80% confluence. Following the manufacturer’s protocols and our previous study [45], we co-transfected 400 ng reporter plasmids and 20 ng pRL-TK into HEK293T cells. This transfection reagent did not require medium replacement after transfection. Based on our recent studies [46], two Cu concentrations, namely the control (without extra Cu addition) and Cu-treated group (10 μM Cu), were used in this experiment, and Cu was added in the form of CuSO_4_·5H_2_O. After the incubation for 24 h, the cells were lysed and collected for analyzing the relative luciferase activities by the Dual-Luciferase Reporter Assay System (Promega, Madison, WI, USA). The relative luciferase activities were obtained via the calculation of the ratio of Firefly to Renilla luciferase activity. We performed each cell transfection in triplicate, and the experiments were repeated in triplicate independently.

#### 4.3.4. Site-Mutation Analysis of NRF2 and SREBP1 Binding Sites on ctr1, ctr2 and atox1 Promoters 

To identify the NRF2 and SREBP1 binding sites on yellow catfish *ctr1, ctr2* and *atox1* promoters, we used the QuickChange II Site-Directed Mutagenesis Kit (Vazyme, Piscataway, NJ, USA) to perform the site-mutation analysis. The pGl3-ctr1-1359, pGl3-ctr2-1842 and pGl3-atox1-1825 were used as the templates. The site-mutation primers are presented in Table S3. The mutant plasmids were named as Mut-ctr1-NRF2(1), Mut-ctr1-NRF2(2), Mut-ctr1-NRF2(12), Mut-ctr1-SREBP1(1), Mut-ctr1-SREBP1(2), Mut-ctr1-SREBP1(3), Mut-ctr1-SREBP1(12), Mut-ctr1-SREBP1(13), Mut-ctr1-SREBP1(23), Mut-ctr1-SREBP1(123), Mut-ctr2-SREBP1(1), Mut-ctr2-SREBP1(2), Mut-ctr2-SREBP1(12), Mut-atox1-NRF2(1), Mut-atox1-NRF2(2), Mut-atox1-NRF2(3), Mut-atox1-NRF2(12), Mut-atox1-NRF2(13), Mut-atox1-NRF2(23), Mut-atox1-NRF2(123), Mut-atox1-SREBP1(1), Mut-atox1-SREBP1(2) and Mut-atox1-SREBP1(12), respectively. Using Lipofectamine 293 (Beyotime Biotechnology, Shanghai, China), we co-transfected these mutant plasmids and pRL-TK into the HEK293T cells. This transfection reagent did not require medium replacement after transfection. Two Cu concentrations, such as the control (without extra Cu addition) and Cu-treated group (10 μM Cu), were used in this experiment. After 24 h of incubation, the cells were lysed and collected for analyzing the relative luciferase activities.

#### 4.3.5. Primary Hepatocyte Culture and Treatments 

According to Wu et al. [47], we isolated and cultured yellow catfish primary hepatocytes. Based on Chen et al. [46], we designed the control (without extra Cu addition) and Cu-treated group (10 μM Cu). After 48 h incubation, the cells were collected for electrophoretic mobility shift assay (EMSA).

#### 4.3.6. EMSA 

The EMSA experiment was performed to identify the functional NRF2 and SREBP1 binding sites of ctr1, ctr2 and atox1 promoters. After the incubation with or without 10 μM Cu for 48 h, the nucleus proteins were extracted from primary hepatocytes of yellow catfish. Then, we used the BCA method to determine the protein concentrations. The Lightshift Chemiluminescent EMSA kit (Beyotime Biotechnology, Shanghai, China) was used in our study. Briefly, we first incubated the oligonucleotide duplexes of NRF2 and SREBP1 with 10 μg nucleus proteins at room temperature for 10 min. The biotin probe was added and incubated at room temperature for another 20 min. Subsequently, the loading buffer was added, and the electrophoresis was performed on a 6.5% native polyacrylamide gel. After the electrophoresis, the bands were transferred to nitrocellulose films. By blocking and washing together with HRP (1:2000), we used the Vilber Fusion FX6 Spectral imaging system (Vilber Lourmat) to visualize the binding bands. We also used 100-fold excess of unlabeled double-stranded oligonucleotides, in combination with or without the NRF2 and SREBP1 mutation, for the competitive analyses. Table S4 lists all the oligonucleotide sequences for EMSA.

### 4.4. Exp. 2: Effects of Dietary Cu Levels on Ctr1, Ctr2 and Atox1 Transcriptional Responses in Yellow Catfish Liver Tissues 

#### 4.4.1. Animals’ Feeding, Management and Sampling 

The experimental protocols for feed formulation, animal culture and feeding were based on Zhao et al. [48] and Zhong et al. [31]. Dietary Cu was added in the form of CuSO_4_·5H_2_O at the levels of 0.008 (adequate Cu, AC) and 0.4 (Cu excess, CE) g/kg diet, respectively. Referring to Tan et al. [49], the Cu content in the AC diet meets the dietary Cu requirements of yellow catfish. 180 juvenile yellow catfish (3.5 ± 0.01 g, mean ± SD) were stocked in six tanks (300 L water volume), with 30 fish per tank. They were fed two full meals a day. The feeding experiment lasted for 10 weeks. 

At the end of the feeding experiment, yellow catfish were fasted for 24 h to avoid postprandial effects, euthanized with MS-222 (Sinopharm Chemical Reagent Co., Ltd., AE1052101) solution, and then sampled. Nine fish were randomly selected from each tank, and their liver samples were taken, quickly frozen in liquid nitrogen, and stored at −80 °C for real-time quantitative PCR (qPCR) and Western blot analysis, respectively.

#### 4.4.2. qPCR

The experimental protocols for the qPCR assay were similar to those described in Wu et al. [47]. We selected 10 housekeeping genes, namely *β-actin*, tubulin alpha chain (*tuba*), TATA-box-binding protein (*tbp*), *18srRNA*, translation elongation factor (*elfa*), beta-2-microglobulin (*b2m*), glyceraldehyde-3-phosphate dehydrogenase (*gapdh*), ribosomal protein L7 (*rpl7*), hypoxanthine-guanine phosphoribosyltransferase (*hprt*) and ubiquitin-conjugating enzyme (*ubce*), to analyze their expression stability. We used the online tool geNorm (https://genorm.cmgg.be/) to select the most stable two genes for endogenous control. Then, the 2^−ΔΔCt^ method was used to calculate the gene mRNA abundances. The PCR primers for each gene are given in Table S5.

#### 4.4.3. Western Blot Analysis

Based on our recent experimental protocols [50], we performed Western blot analysis to determine the expression of these proteins. The specific primary antibodies included anti-CTR1 (1:750, 27499-1-AP; Proteintech Group, Wuhan, China), anti-CTR2 (1:1000, A16362; Abclonal, Wuhan, China), anti-ATOX1 (1:1000, A9874; Abclonal, Wuhan, China) and anti-GAPDH (1:10,000, 10494-1-AP; Proteintech Group, Wuhan, China). The Vilber Fusion FX6 Spectra imaging system (Vilber Lourmat) was used to visualize these protein bands, followed by the quantitative analysis using Image-Pro Plus 6.0.

### 4.5. Exp. 3: Intracellular Co-Location Analysis of Ctr1, Ctr2 and Atox1

#### Immunofluorescence

Based on Pang et al.’s study [51], we constructed the open reading frame of CTR1, CTR2 and ATOX1 with the deletion of stop codons into vector pcDNA3.1-EGFP, respectively. Then, fusion plasmids were transfected into HEK293T cells. After 24 h incubation, the cells were fixed in the 4% paraformaldehyde. Subsequently, Dil (Beyotime Biotechnology, Shanghai, China) was used as a red fluorescent dye for cell membrane staining, Hochest (Beyotime Biotechnology, Shanghai, China) was used as a blue fluorescent dye for cell nucleus staining, and Lyso-Tracker Red (Beyotime Biotechnology, Shanghai, China) was used as a red fluorescent dye for lysosome staining, respectively. We visualized these images using laser confocal microscopy (Leica, Carl Zeiss, Jena, Germany).

### 4.6. Statistical Analysis

We used the SPSS 22.0 software (Armonk, NY, USA) to perform the statistical analysis. All the data were presented as means ± standard errors of means (SEM). The Student’s *t*-test was used to analyze the data between two treatments, and one-factor ANOVA and Duncan’s multiple range test were used to analyze the data among ≥ 3 treatments. The *p* < 0.05 was considered to be statistically significant.

## 5. Conclusions

In conclusion, we identified the promoters of the Cu-transport-related genes *ctr1*, *ctr2* and *atox1* in yellow catfish, and identified functional NRF2 and SREBP1 binding sites in these three promoters. Cu could regulate the activities of these three promoters through NRF2 and SREBP1. Dietary Cu addition influenced the *ctr1*, *ctr2* and *atox1* mRNA and total protein levels, thereby maintaining Cu homeostasis. We also determined the intracellular localization of CTR1, CTR2 and ATOX1. Taken together, our study provided new evidence for the transcriptional response mechanism of *ctr1*, *ctr2* and *atox1* promoters to NRF2 and SREBP1 response elements, and provided new ideas for the role of Cu transporter-related proteins CTR1, CTR2 and ATOX1 in controlling Cu homeostasis in vertebrates.

## Data Availability

The data used to support the findings of this study are available from the corresponding author upon reasonable request.

## Supplementary Materials

The following supporting information can be downloaded at: https://www.mdpi.com/article/10.3390/ijms232012243/s1.

## Abbreviations

AC: Adequate Cu; AP1, Activator proteins 1; ARE, Antioxidant response element; ATOX1, Antioxidant-1 copper chaperone; ATP7A, ATPase copper transporting alpha; ATP7B, ATPase copper transporting beta; *b2m*, Beta-2-microglobulin; CCS, Copper chaperone for superoxide dismutase; CE, Cu excess; COX17, Cytochrome c oxidase copper chaperone; CREB1, cAMP-response element-binding protein 1; CTR1, Copper transporter 1; CTR2, Copper transporter 2; Cu, Copper; Dil, 1,1’-dioctadecyl-3,3,3’,3’-tetramethylindocarbocyanine perchlorate; DMEM, Dulbecco’s Modified Eagle’s Medium; DMT1, Divalent metal ion transporter 1; *elfa*, Translation elongation factor; EMSA, Electrophoretic mobility shift assay; FBS, Fetal bovine serum; *gapdh*, Glyceraldehyde-3-phosphate dehydrogenase; *hprt*, Hypoxanthine-guanine phosphoribosyltransferase; *klf4*, Kruppel-like factor 4; MAC1, Metal-binding activator 1; MRE, Metal-responsive element; MS-222, 3-aminobenzoic acid ethyl ester methanesulfonate; NF-Y, Nuclear factor Y; NRF2, Nuclear factor erythroid 2-related factor 2; PPARγ, Peroxisome proliferator-activated receptor gamma; qPCR, Real-time fluorescence quantitative PCR; *rpl7*, Ribosomal protein L7; SEM, Standard error of means; SP1, Specifificity protein 1; SREBP1, Sterol regulatory element-binding proteins 1; STAT3, Signal transducer and activator of transcription factor 3; STAT4, Signal transducer and activator of transcription factor 4; *tbp*, TATA-box-binding protein; TFBS, Transcription factor binding sites; TSS, Transcription start site; *tuba*, Tubulin alpha chain; *ubce*, Ubiquitin-conjugating enzyme.
