# Supplementary material for: Transcriptional Responses of Copper-Transport-Related Genes ctr1, ctr2 and atox1 and Their Roles in the Regulation of Cu Homeostasis in Yellow Catfish Pelteobagrus fulvidraco
Source: Int J Mol Sci. 2022 Oct 13;23(20):12243. doi: 10.3390/ijms232012243 (PMC9602723; doi:10.3390/ijms232012243)
Supplement: Supplementary file 1 [file ijms-23-12243-s001.zip › File S1.pdf]

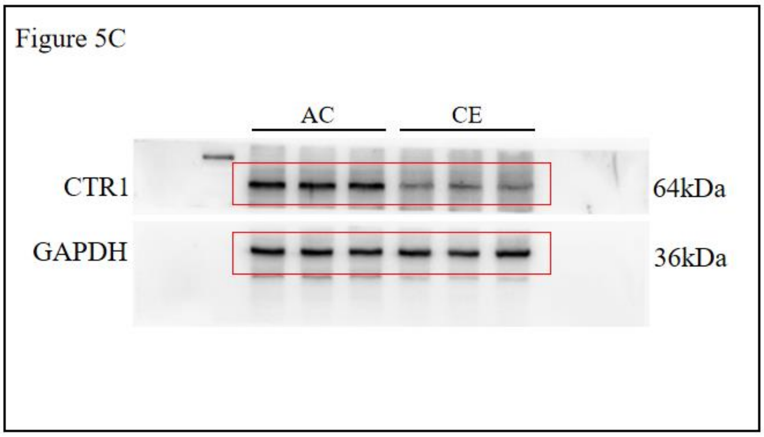

Western Blotting protein original Figure 5C.
Full scans of uncropped blots presented in the paper. Red boxes indicate the cropped regions. Molecular weight is indicated in kDa.

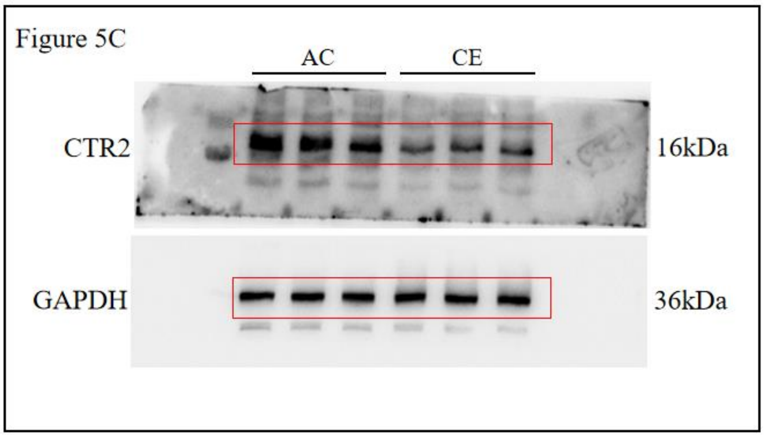

Western Blotting protein original Figure 5C.
Full scans of uncropped blots presented in the paper. Red boxes indicate the cropped regions. Molecular weight is indicated in kDa.

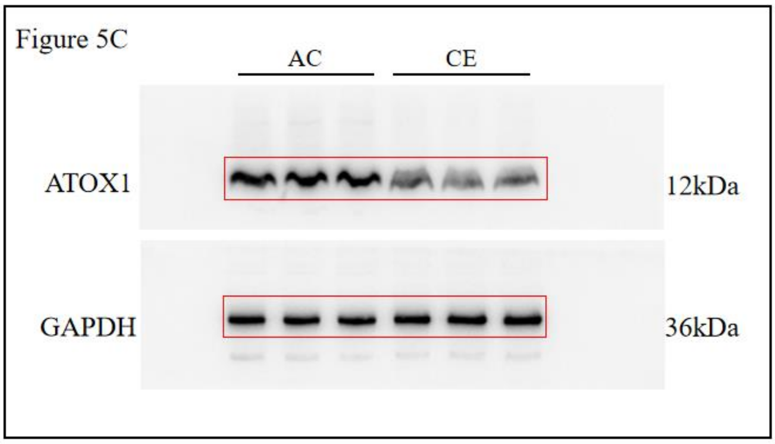

Western Blotting protein original Figure 5C.
Full scans of uncropped blots presented in the paper. Red boxes indicate the cropped regions. Molecular weight is indicated in kDa.

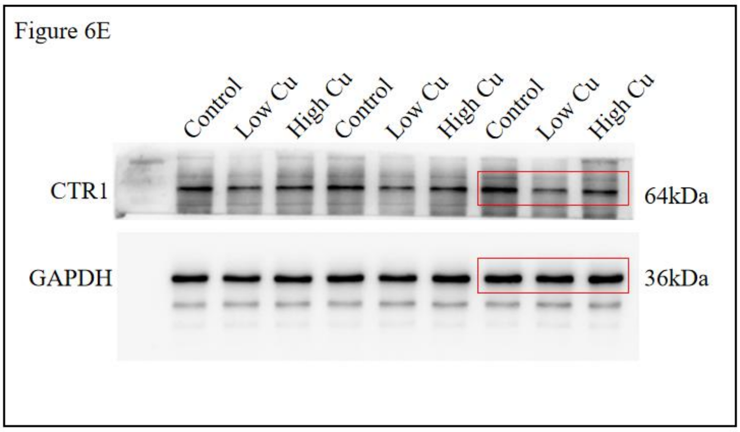

Western Blotting protein original Figure 6E.

Full scans of uncropped blots presented in the paper. Red boxes indicate the cropped regions. Molecular weight is indicated in kDa.

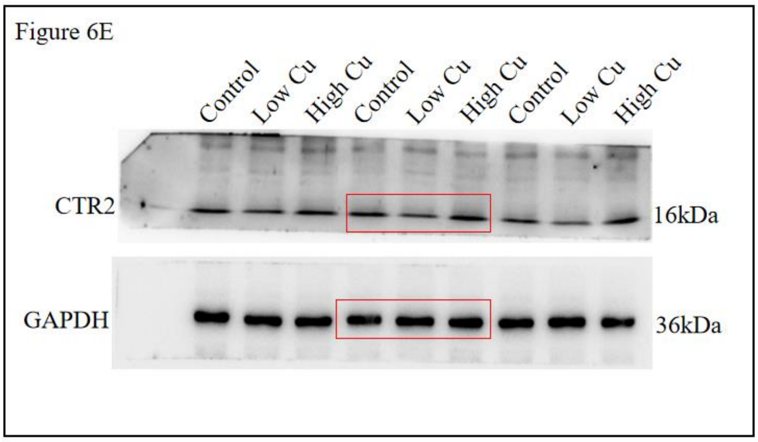

Western Blotting protein original Figure 6E.

Full scans of uncropped blots presented in the paper. Red boxes indicate the cropped regions. Molecular weight is indicated in kDa.

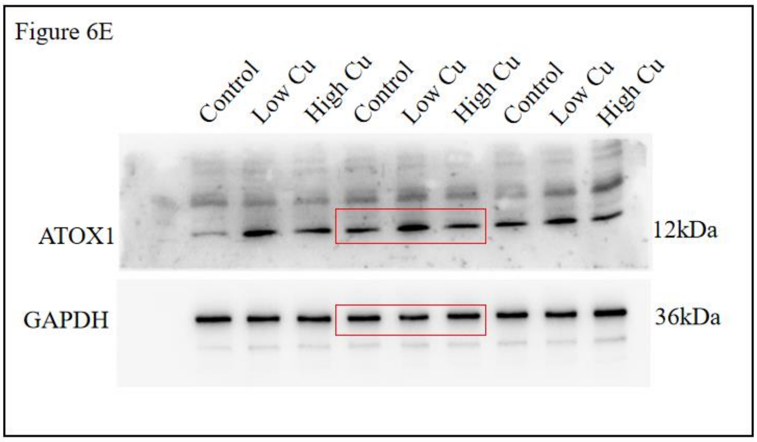

Western Blotting protein original Figure 6E.

Full scans of uncropped blots presented in the paper. Red boxes indicate the cropped regions. Molecular weight is indicated in kDa.

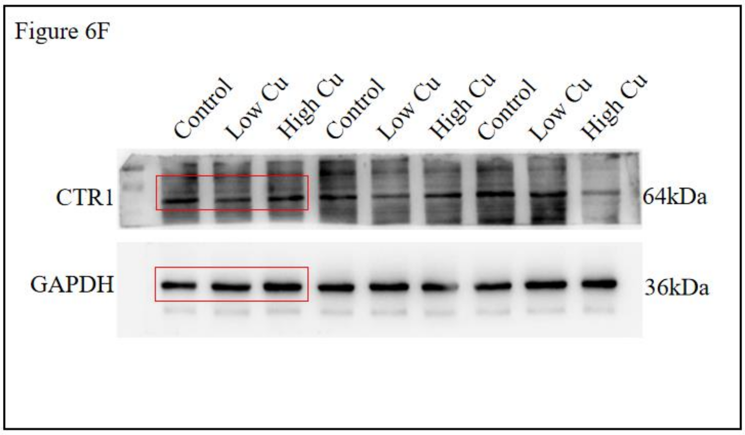

Western Blotting protein original Figure 6F.

Full scans of uncropped blots presented in the paper. Red boxes indicate the cropped regions. Molecular weight is indicated in kDa.

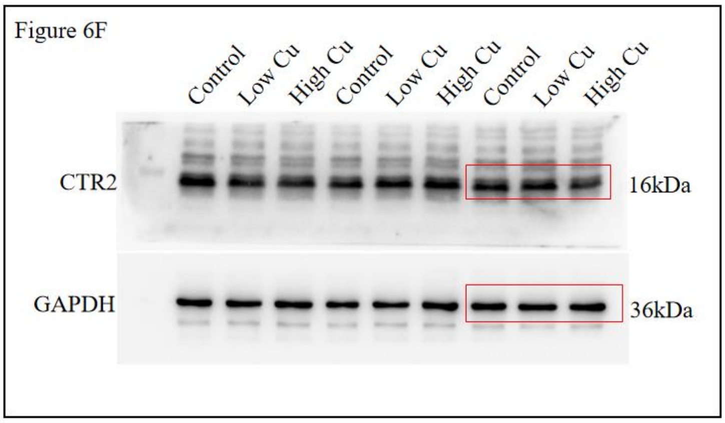

Western Blotting protein original Figure 6F.

Full scans of uncropped blots presented in the paper. Red boxes indicate the cropped regions. Molecular weight is indicated in kDa.

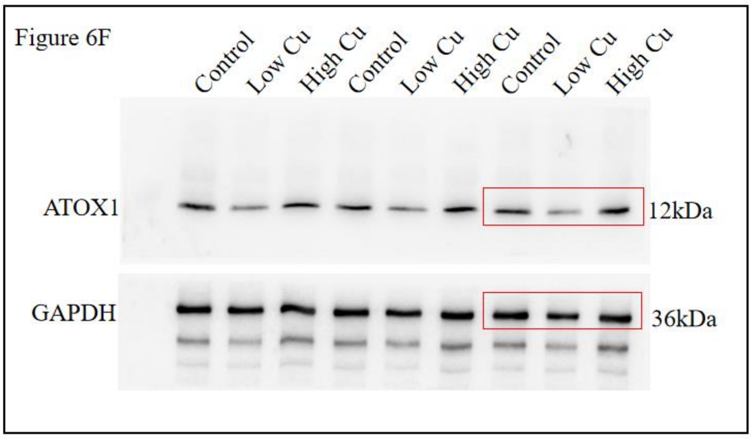

Western Blotting protein original Figure 6F.

Full scans of uncropped blots presented in the paper. Red boxes indicate the cropped regions. Molecular weight is indicated in kDa.
