# Supplementary material for: Transcriptional Responses of Copper-Transport-Related Genes ctr1, ctr2 and atox1 and Their Roles in the Regulation of Cu Homeostasis in Yellow Catfish Pelteobagrus fulvidraco
Source: Int J Mol Sci. 2022 Oct 13;23(20):12243. doi: 10.3390/ijms232012243 (PMC9602723; doi:10.3390/ijms232012243)
Supplement: Supplementary file 1 [file ijms-23-12243-s001.zip › Supplementary tables and figures.pdf]

Table S1. Primers used for the cloning of *ctr1*, *ctr2* and *atox1* promoters in yellow catfish.

Genes	Forward primer (5'-3')	Reverse primer (5'-3')
ctr1	TGTGGAGCGACTTGTGTGTGGG	GACGAGGTCCTGACGGCTACCGGC
ctr2	CCCAAACGCAATTAAAAATGTTGA	TTATTATATAATCCAGCTACAGCTT
atox1	TACAAATATTAACCAGTATTTA	AAGAAGAAGAAAAAAAGACACT

Table S2. Primers used for 5'-deletion plasmids construction of *ctr1*, *ctr2* and *atox1* promoters in yellow catfish

Genes	Primers	Forward primer (5'-3')	Reverse primer (5'-3')
ctr1	pGl3-470/+57	ctatcgataggtaccgagctcGACTACTTACTATGTCCTTAGCCCTGTC	cagtaccggaatgccaagcttGACGAGGTCCTGACGGCTACC
	pGl3-796/+57	ctatcgataggtaccgagctcTCCACAGAAGCAGTTTCAGTCATT	cagtaccggaatgccaagcttGACGAGGTCCTGACGGCTACC
	pGl3-1039/+57	ctatcgataggtaccgagctcAGTGCTGTGGTGCTGGTGAAG	cagtaccggaatgccaagcttGACGAGGTCCTGACGGCTACC
	pGl3-1359/+57	ctatcgataggtaccgagctcTGTGGAGCGACTTGTGTGTGG	cagtaccggaatgccaagcttGACGAGGTCCTGACGGCTACC
ctr2	pGl3-522/+43	ctatcgataggtaccgagctcGTCAAATTGCCGAAATACTCCAT	cagtaccggaatgccaagcttTTATTATATAATCCAGCTACAGCTTTGTCC
	pGl3-1019/+43	ctatcgataggtaccgagctcCAACAGGCAGCAGCACCATA	cagtaccggaatgccaagcttTTATTATATAATCCAGCTACAGCTTTGTCC
	pGl3-1557/+43	ctatcgataggtaccgagctcCGTCACTCTGTACTCAGGGAAGG	cagtaccggaatgccaagcttTTATTATATAATCCAGCTACAGCTTTGTCC
	pGl3-1842/+43	ctatcgataggtaccgagctcCCCAAACGCAATTAAAAATGTTG	cagtaccggaatgccaagcttTTATTATATAATCCAGCTACAGCTTTGTCC
atox1	pGl3-649/+43	ctatcgataggtaccgagctcTCAGCTAGTTCAGCGTGCAGAT	cagtaccggaatgccaagcttAAGAAGAAGAAAAAAAGACACTCGG
	pGl3-1088/+43	ctatcgataggtaccgagctcCATTGACCTGCATATGGTTCATG	cagtaccggaatgccaagcttAAGAAGAAGAAAAAAAGACACTCGG
	pGl3-1592/+43	ctatcgataggtaccgagctcGAAAGTTGGATCAAAATATATCACAAATC	cagtaccggaatgccaagcttAAGAAGAAGAAAAAAAGACACTCGG
	pGl3-1825/+43	ctatcgataggtaccgagctcTACAAATATTAACCAGTATTTATATGACAGTGG	cagtaccggaatgccaagcttAAGAAGAAGAAAAAAAGACACTCGG

Table S3. Primers used for site-mutation analysis

Genes	Primers	Forward primer (5'-3')	Reverse primer (5'-3')
ctr1	Mut-ctr1-NRF2(1)	gttgacagtcagtagcagcATAACAGGGCCGTGTTATTAAAACA	ctgctactgactgtcaacattACACACCTGCTAACCTGGAGTGT
	Mut-ctr1-NRF2(2)	aaatcagaagcctggtgtgagGAACAGTGCTGTGGTGCTGGT	caccaggcttctgatttgcaaGTTACAGGTAATATGACATCTATACAATATTAAAC
	Mut-ctr1-SREBP1(1)	atcccagtggtcacagtttagTCACGAGGGATCGTTTTTTTTT	aactgtgaccactgggatgtggaGCGACTTGTGTGTGGGTGACC
	Mut-ctr1-SREBP1(2)	ccctccttgataagtcaattaagCCAGACGATGTAACTCTATTCTTATTTCC	tgacttatcaaggagggAGAAAGATCACATGACCAGTTTAGTCA
	Mut-ctr1-SREBP1(3)	accgacatagcgattgtcTGAACTTACAGTATTTACACACGGTATTG	gacaatcgctatgtcggtaataCTCGAATGAACAGTTTCTTAGCAGC
ctr2	Mut-ctr2-SREBP1(1)	agcatcacatcgcactaggtTGCTAAACCGGTAGCCGGC	tagtgcgatgtgatgctcgaAGCTAAACTTGACTAAACTAACTAGCTTTCT
	Mut-ctr2-SREBP1(2)	atgccagatatggcttgatcAGCAACTCCACACAAAATGCC	caagccatatctggcatgtcTGCTGAGGAAAAGTCCAAAATCA
atox1	Mut-atox1-NRF2(1)	tcagtcagagtcggtacggctACAGTCAGTTCAAAGTGACAGAGTTTG	gtaccgactctgactgactTAGGGATGATCGTTAAATTCCCA
	Mut-atox1-NRF2(2)	tcatgacaggctaggtgtccACAGGCAACGGAGCTTCAAC	cacctagcctgtcatgattcGGTTCACAGAAGCACAAACTATAGCG
	Mut-atox1-NRF2(3)	gcttgtcagagtcatgtataaaCAGTTTCTTCCATGACTGACGTTT	acatgactctgacaagccACAGTGATCAGCAGTTTCTTGTAGTG
	Mut-atox1-SREBP1(1)	gaaccgcgtgtcgttttcttCACACAGTATAAATGATAACATATAATGTGTCA	aaaacgacacgcggttcgtaACACTGCCTAGGCGCTTGATT
	Mut-atox1-SREBP1(2)	agccgtcagaggtcgctactcgGTCAGCTGGTACAACTACCAGGG	tagcgacctctgacggctacAAGAAGACTAACAGGGAAAAGCATAA

Table S4. Primers used for electrophoretic mobility-shift assay

Primers		Forward primer (5'-3')	Reverse primer (5'-3')
ctr1-NRF2(1)	Biotin-probe	Biotin-GTAATGTGTGACACAGACAGCAGCA	Biotin-TGCTGCTGTCTGTGTCACACATTAC
	Mutative-competitor	ACGGCTCTGACAGTCAGTGATCATG	CATGATCACTGACTGTCAGAGCCGT
ctr1-NRF2(2)	Biotin-probe	Biotin-TTGCAAAGTGACCAATCAGTGTGAG	Biotin-CTCACACTGATTGGTCACTTTGCAA
	Mutative-competitor	CGATGGCTCAGAAGCCTGACTGACT	AGTCAGTCAGGCTTCTGAGCCATCG
ctr1-SREBP1(1)	Biotin-Probe	Biotin-GAGAAAGATCACATGACCAGTTTAG	Biotin-CTAAACTGGTCATGTGATCTTTCTC
	Mutative-competitor	ACTCGCACCAGTGGTCATGACCGGT	ACCGGTCATGACCACTGGTGCGAGT
atox1-NRF2(2)	Biotin-probe	Biotin-CGAATCAGTCACAAAGCAGTGTCCA	Biotin-TGGACACTGCTTTGTGACTGATTCG
	Mutative-competitor	ATGCGACTGACAGGCTAGTCACTAG	CTAGTGACTAGCCTGTCAGTCGCAT
atox1-NRF2(3)	Biotin-probe	Biotin-TGGCTTGCTGAGACAGCATATAAAC	Biotin-GTTTATATGCTGTCTCAGCAAGCCA
	Mutative-competitor	CATAGGATCAGAGTCATGCGGCCGA	TCGGCCGCATGACTCTGATCCTATG
atox1-SREBP1(1)	Biotin-probe	Biotin-TTACGAAATTAAGTGATTTTTCTTC	Biotin-GAAGAAAAATCACTTAATTTCGTAA
	Mutative-competitor	CGCATCGCCGCGTGTCGGCCGAGCT	AGCTCGGCCGACACGCGGCGATGCG
atox1-SREBP1(2)	Biotin-probe	Biotin-GTAGCCGGTCACATGATCTACTCGG	Biotin-CCGAGTAGATCATGTGACCGGCTAC
	Mutative-competitor	ACGTATATCAGAGGTCGTGGACATA	TATGTCCACGACCTCTGATATACGT

Table S5. Primers used for RT-qPCR analysis

Genes	Forward primer (5'-3')	Reverse primer (5'-3')	Accession No.
ctr1	TCAACACACCTGGAGAGATG	CACCTGGATGACGTGAAGTA	KY646156
ctr2	CAAGGTGTGGAAGAACGTTC	GAACAATGTGCAGTCCTGTC	KY646157
atox1	TGACATGTGAAGGATGCTCT	AGGACCAATATACGTGACAGT	KY660270
β-actin	GCACAGTAAAGGCGTTGTGA	ACATCTGCTGGAAGGTGGAC	EU161066
elfa	GTCTGGAGATGCTGCCATTG	AGCCTTCTTCTCAACGCTCT	KU886307
b2m	GCTGATCTGCCATGTGAGTG	TGTCTGACACTGCAGCTGTA	KP938520
hprt	ATGCTTCTGACCTGGAACGT	TTGCGGTTCAGTGCTTTGAT	KP938523
rpl7	GGCAAATGTACAGGAGCGAG	GCCTTGTTGAGCTTGACGAA	KP938522
ubce	TCAAGAAGAGCCAGTGGAGG	TAGGGGTAGTCGATGGGGAA	KP938524
18s rRNA	AGCTCGTAGTTGGATCTCGG	CGGGTATTCAGGCGAGTTTG	KP938527
tbp	AGCAAAGAGTGAGGAGCAGT	ACTGCTGATGGGTGAGAACA	KP938525
gapdh	TTTCAGCGAGAGAGACCCAG	ATGACTCTCTTGGCACCTCC	KP938521
tuba	TCAAAGCTGGAGTTCTCGGT	AATGGCCTCGTTATCCACCA	KP938526

```
TGTGGAGCGA CTTGTGTGTG GGTGAGAGAG TGCTTGTGAG AGTGTTTGTG TGTAAGAGTG TATGTGGAAA AGAGTGTGTG GAAGAGAGTA TTTGTGTGTG -1259
                                                                           KLF4
TGATAGAGAG AGTGCACACG TAAGTGTGTG CTTGAACAAA AGACTACACG ATTTATAGTT ATAGAGTTAT AGTTCATGTC GGCCATGTTT GTAGGCCGTG -1159
              MRE            MAC1
ATGTTCTCTG TGAAATTGAG TCAAACTGAT ATTTTTAAGT TAATCATTTC TTAGTTCTGT TTAATATTGT ATAGATGTCA TATTACCTGT AACTTGCAAA -1059

GTGACCAATC AGTGTGAGGA ACAGTGCTGT GGTGCTGGTG AAGTAAATCC AGTTATGAAG CTGTTTGGAG TTGTTGAGAA GAAAACGGTG TTGTAGCAGA -959
  NRF2                               CREB1
GCAGGTATAA TTTATTCTCC CTGGTTACCA TACTGTAGTA AACCGAACAT CGTGTTTATC ATCATATATT AAATAACTCA GATCCAGTCC CACACTCCAG -859

GTTAGCAGGT GTGTAATGTG TGACACAGAC AGCAGCATAA CAGGGCCGTG TTATTAAAAC ATTTTCCACA GAAGCAGTTT CAGTCATTCT GCTCATACTG -759
                        NRF2                                                                     MAC1
TACATTTAAG TTGTTGCTGT TTTTTGCACA ATTTTATACA CTATCTCAGG GACCTGCTGC TAAGAAACTG TTCATTCGAG TATTACTTCA CGCAATATTG -659
                        MAC1                               STAT3                          SREBP1
TCTGAACTTA CAGTATTTAC ACACGGTATT GACGCCGGTC GGTCGGCGCT GTTTTTTGTT TACTGTCTTT TTTGTATTTT GTGTGTTGTC TTGTAACCTT -559
                MRE
TTGTCTGAAC TGTCTTGTCT GTCTTGTCCT GCACTGTCTT GTCTTGCCCT GTTTGCACCA GGTCACACAG TTGCACTTTA TGTATCTAGG ACTACTTACT -459

ATGTCCTTAG CCCTGTCTTT GTTTAATGTA GCACCTTGAT CCTGGAGAAA CGTCGTCTCA TTTCACTTTG TACTGTAACA GCTATATATG GTTGAAATGA -359

CAATAAAAGC TTCTTGACTT GACTTGAAAA AGGTTTCTCT GACAGGGGTC AGGAGTAGCG CGACACAGGC ACTGGTCTCA AATCGGTCAT TAACACTTTA -259
                                                            AP1
CCAAGCAATA CTAAGTAATT AGAAATGTGT GATAATGATA TAGTCCCTGT CAGATGAATC CTTACATTCC TTCATCAATC AAATAAGGGA AATAAAGAAA -159

AGGAAATAAG AATAGAGTTA CATCGTCTGG CTTAATCTCA GCCCAGAGGA GGGAGAAAGA TCACATGACC AGTTTAGTCA CGAGGGATCG TTTTTTTTTT -59
                         CCAAT-box    SREBP1       SP1          SREBP1
TTAAAGGTCA CCCACACACA AGTCGCTCCA CATCTGCACA CACCCAAGAG CCAAGTTCCA TTTCTCGGTG TAAAGTACCG CTTCACGTGC GCGCCGGTAG +41
 TATA-box                             MRE                    TSS
CCGTCAGGAC CTCGTC +57
```

Figure S1. Nucleotide sequence of yellow catfish *ctr1* promoter. Numbers are relative to the transcription start site (TSS) (+1). The putative transcription factor binding sites are underlined. The highlighted sequences show putative transcription factor binding sites of NRF2 and SREBP1.

```
CCCAAACGCA ATTAAAAATG TTGAGTTAAG TGTGGAAAAA CCCTTAAGTT TGACATTTTA TCTTTTCTCC TGTTAAACAC TATTGTAATG CTGCGTTCAT -1742

GCTACCTAGT AAACAGGACG AGGAGTTATA AACTCAAAAT AGCAAGGAGT ATAGCTGGTG TATTAGATCG AACTAAATAC TGTCTGTATA ATAACTGATC -1642

TAAAACGAAT ACTGTTAGAA ACTAATTTAA AAGTAGAGAA GGGGGACTTT AGTCCATTTT TCACTGGGTG TGACCAAGAG GATTTGACGT CACTCTGTAC -1542
                                                                     KLF4                              MAC1
TCAGGGAAGG AGCTGGTAGC GCCAATGACT ACCTCAAGAA GCTAGCAGGT CAAAATTACT AGTTAAAGTG AGGTTCCACT GTGTGTGTGA GTGTGTGTGT -1442

TGTTTTTTTT CAGTAATAGC GCAATTACAT GTTCGCCTCG AAAACAAGAG CACAAAGAAC ATCACCTTGT GATGTCAGTG ACATACTTCT CACAGGAATA -1342
                                                 MAC1                CREB1
ACAAATGTAC ATCACTAAAA ATGAATTAAA CCCCAAATAG ATGGAAGACA ACATGTTTTT TTTTTCAATA TATATTTAAA GCTGTTCAAT CAGAGGCTGG -1242
                                                                                                          SP1
ATGTTAATTA ATGTGTTTTA ATTAACATAT TTTACTTCTT GATACTGCCA TGGTTCTATA GCTGTGAACA GACTGATGAG GCTCATGGGG CTCAGTTTGT -1142

AATCGAATGA TACTAAATAA AAAATAGTGA TGGATACAAA AGTACCTGAT TTTGGACTTT TCCTCAGCAG ACATGATCTG GCCATCTTGA TCAGCAACTC -1042
                                                           PPARγ              SREBP1
CACACAAAAT GCCACTAAAG AGCACAACAG GCAGCAGCAC CATAACCGAG CAGCTGAAGC AGGGACTCTT CTCTGCTCTT ACACCTATTT TACCCATTAA -942

AACTAGATTT TTCCCCCTAC TGATCGTGAA AGGAAAACGA CTTTTTTTCC TTCCAAGAGA TCCTGAACTG TCTTTAATTA AAGTACACGT AAACAGTTAA -842
                          STAT4
AGCATGATAG TAAACCTCCT CCTGTCAGTC AAGGAACAAT TAATAAACAA CACGGAAATA AACAACCACA AGCTTTGAAT AGCCAAATAT TTTAAACGAC -742

TCAAACAATT AGTGTAAATT ACGACAGGTA GATAACACGT ATATCTAACC TCGCCTAAGC TAAACAGAAA GCTAGTTAGT TTAGTCAAGT TTAGCTTCGA -642

GCAGTGACGT TAGCTAGGTT GCTAAACCGG TAGCCGGCTA AGCTAACTAC TTCATAGCTA ACACAAGAAA AGAGAAAAGT CCTTCTTGGA TAAATATTCC -542
   SREBP1                                                                                              STAT3
AGAAAATGTT CCCTGGAGGA GTCAAATTGC CGAAATACTC CATTTCCAAC CGTCCTCAGA TGTACATATT TTCGTGAAGG CTCATCTAAA CCCAGCGACT -442

CCTCGCCGCC TCCTGTCTAA TCAAAACACC AAAATATTCA CATATTTACA TTGACGCCAT CTTTCTTTAC ACTCCGACAA GCCGGAAGTA GAATTAAAAA -342
   SP1                                                                                    AP1
TAATATATAT TTTTATGATT TTTAAACACA CGATAAAAGG CATTTTCATG TCACATTACA TTTCATGTCA CATTTTTATA TTATTATGTT ATATAAAAAG -242

ATTTATATAT ATAAAAAAAA TAAAAAAGTA CTGTATGAGT AACCAATGAC AGCTCAAATT TGAGTCAGTG TGACGTATTT TAACGGACGA ATCCAAATAT -142
                                              CCAAT-box             CREB1
CACTTTAGTG CACTAGGTAG GGTACAGGAG AAGCTTTTGG TTATTCACTA CCTGGTAGAG CTAGTCTAGG GAGTAGGGAA CAATTTGGTT TTTACTGCAT -42
                     PPARγ
TTGCACGTTT ACTTATATAG CTTGTTTCCC TTTGTGAAAC CGTCTAGGAG CAGGAGGACA AAGCTGTAGC TGGATTATAT AATAA +43
        TATA-box                           TSS
```

Figure S2. Nucleotide sequence of yellow catfish *ctr2* promoter. Numbers are relative to the transcription start site (TSS) (+1). The putative transcription factor binding sites are underlined. The highlighted sequences show putative transcription factor binding sites of SREBP1.

```
TACAAATATT AACCAGTATT TATATGACAG TGGGTTGGGT CCCTGGTGGT CCAATAGCAG GATCTCACTG CCACAGTCAG GGTTCGATTC CCAGGCAGGG -1725
                                    SP1
AACAAACTCA GACACTGAGG GGTTCATTGC TGGTCCTTAG GAAGGGAATC CAGCATAAAA CCTGCGCCAA ATTAAATATA GGTGGATCGA ATGATTTGCT -1625
                                                                      AP1
GTGGTGAAAG AGGCAAGAGC AATATTCATA TGACAGAAAG TTGGATCAAA ATATATCACA AATCTTATCT AGTATTTCAG AATAAAAACA GCTCTGTTAT -1525
              MAC1
CAATGCATGA CTTCCTTAAA TGACTCTAAG CATTTACTGA AGGCTATTTA AACATCTACA ATTAACTTGT TGAACTATAT GATATACATG TCATCATGAG -1425
                                                                                                 CREB1
TGATTACAGT ACAATTGTGT TGCTTTTTTC AGGGCGTAAA AGGCATACGT TAACCATGAA ATTGTGCAAG TGAAATTAAA AATTACATAT GTAAAATATG -1325

TTATGCAACA GTTAAAAAAT ATGAGGTTTT TTTTTGTTTT GTTTTTTGCC ATGCACTACA AGAAACTGCT GATCACTGTG GCTTGCTGAG ACAGCATATA -1225
                                                                                                NRF2
AACAGTTTCT TCCATGACTG ACGTTTTCGT CCATGACATA ACTATGTTTA ATGAAAAAAA TCTTCTCTTT CATTCAAGGA ATGATGAATC CAACGCTTTC -1125
                                    CREB1
TTTACAGTGA AAACAACAGC ACATTAACAC ATTCAGATCA TTGACCTGCA TATGGTTCAT GTTTCTGTGT TTCACAACAT CTACCATCTA CAGTTATTAT -1025

TATTATACTA TTATTATTAG TTTGAATACG TTCCCACAAA ATAATGAGTC GCTCTTATTT CTTGAAGGCA TCAGTAAATA AACCGGATCT TACTGTGAAA -925
                                                                                       AP1
CCTGCATTGA TTATATCAGA CTTACCAAGG CCGAGAGCAG CTGTAGTCCA ATCTTATTAT GTAACTAGCT TCAAATATTA CAGAAAACTG CACATTTTTA -825

CATGCATTGT TAATCAGATG TTGTTTTATT AAAACCCTGA ACGCTGACCA TCTCATTCCA CTACGAGCCC TTGTCTTTTC CTCCTTATGC TTTTCCCTGT -725
                                                                      KLF4                   KLF4
TAGTCTTCTT GTAGCCGGTC ACATGATCTA CTCGGTCAGC TGGTACAACT ACCAGGGAGC GTCTGTGTAA AACCTGTCAG CTAGTTCAGC GTGCAGATTT -625
                SREBP1
GTTGACTATA TGCTTCCAAA ATACTTCGGA ACTCAAGTGT TTCAGCTGGT TTTGGATAAT AAAAAATAAA ACGCAAACGT GTAATGCTGT ACAAATGTGT -525

GACAACAACA ACAACAACAA AAAATGAGTT AAAATGACAT TTATAGTGAA TTGTTTTTAT TGAATCGACT CGTAAAATAT GAGGATTTGT TTGTGGTTGT -425

TGTTTTTGCC AGTGAAACTA TTTAGAACAT GTTCTTATAC GCAAACAAAC CGCTGTTTCC GCTATAGTTT GTGCTTCTGT GAACCGAATC AGTCACAAAG -325
                                                           AP1                                      NRF2
CAGTGTCCAC AGGCAACGGA GCTTCAACGT CATTGGTCAC GTGTTTATTG CAGAACGAAT CAAGCGCCTA GGCAGTGTTA CGAAATTAAG TGATTTTTCT -225
                                                         CCAAT-box       SP1              SREBP1
TCACACAGTA TAAATGATAA CATATAATGT GTCAATTTAT GCCTGAAAAT ATCTAAAATT ACCGTTCATC AATAATACTT ATATAATAAG CCTAATATTC -125
                                      STAT3                                              TATA-box
ATGTAAACTA AAACATGTTT CTGTTTTCAT AAACGCTTTT GAAAAGTTCT GGGAATTTAA CGATCATCCC TAAGTCAGCA CTGAGTCACA CGGCTACAGT -25
                                                   STAT4                          NRF2
CAGTTCAAAG TGACAGAGTT TGTTATTCAT TTGTGTGTTT TAGCCGAGTG TCTTTTTTTC TTCTTCTT +43
                        TSS
```

Figure S3. Nucleotide sequence of yellow catfish *atox1* promoter. Numbers are relative to the transcription start site (TSS) (+1). The putative transcription factor binding sites are underlined. The highlighted sequences show putative transcription factor binding sites of NRF2 and SREBP1.
